# Identification of the prognostic and immunotherapeutic potential of L antigen family member 3 in malignant pleural mesothelioma

**DOI:** 10.1002/ctm2.207

**Published:** 2020-11-26

**Authors:** Xubin Dong, Jingjing Song, Minzhi He, Jiawen Sun, Endong Tao, Xiaohua Zhang, Quan Li

**Affiliations:** ^1^ Department of Thyroid and Breast Surgery The First Affiliated Hospital of Wenzhou Medical University Zhejiang China; ^2^ Department of Children's Health Care The Second Affiliated Hospital and Yuying Children's Hospital of Wenzhou Medical University Zhejiang China; ^3^ Department of Neonatology The Second Affiliated Hospital and Yuying Children's Hospital of Wenzhou Medical University Zhejiang China; ^4^ Department of Graduate Academy Bengbu Medical College Bengbu China; ^5^ The First Clinical Medical College Wenzhou Medical University Wenzhou China

Dear Editor,

Malignant pleural mesothelioma (MPM) is the most common malignant mesothelioma and difficult to treat. The overall survival period after diagnosis is about 9–17 months, and very few can be cured.^1^ Since high mortality rates of MPM patients do not significantly improve after traditional treatment, immunotherapy is under intensive investigation. Immune checkpoint inhibitors, like programmed death receptor‐1 (PD‐1), its associated ligand (PD‐L1) blockade, and cytotoxic T‐lymphocyte antigen‐4 (CTLA‐4) antibody, showed a disease control in previous clinical trials in MPM.^2^, ^3^, ^4^ The available data confirm that immunotherapy is a feasible treatment for MPM.

L antigen family member 3 (LAGE3) is an intracellular protein that plays a crucial role in regulating positive transcription mediated by RNA polymerase II. It has been shown to affect the accuracy and efficiency of translation and functioned as a component of complex in the metabolism of the tRNA threonine‐carbamoyladenosine process.^5^, ^6^ Previous studies identified LAGE3 was one of the most frequently upregulated RNA modification‐related proteins in multiple cancer types.^7^ Recently, the upregulation of LAGE3 was reported to be correlated with immune cell infiltration and prognosis in clear cell renal cell carcinoma.^8^ Our study is aimed to investigate whether LAGE3 is related to clinical prognosis, immune infiltration, and immunotherapy response for patients with MPM.

In this study, the patients of low‐LAGE3 group provide more durable overall survival (*P* = .003), disease‐specific survival (*P* = .017), and progression‐free interval (*P* < .001) than the MPM patients in high‐LAGE3 group (Figure 1A‐C). Univariate and multivariate Cox regression verified that LAGE3 were independent overall survival (Figure 1D, Table S1) and progression‐free interval (Figure 1E, Table S2) predictive factors in MPM.

**FIGURE 1:**
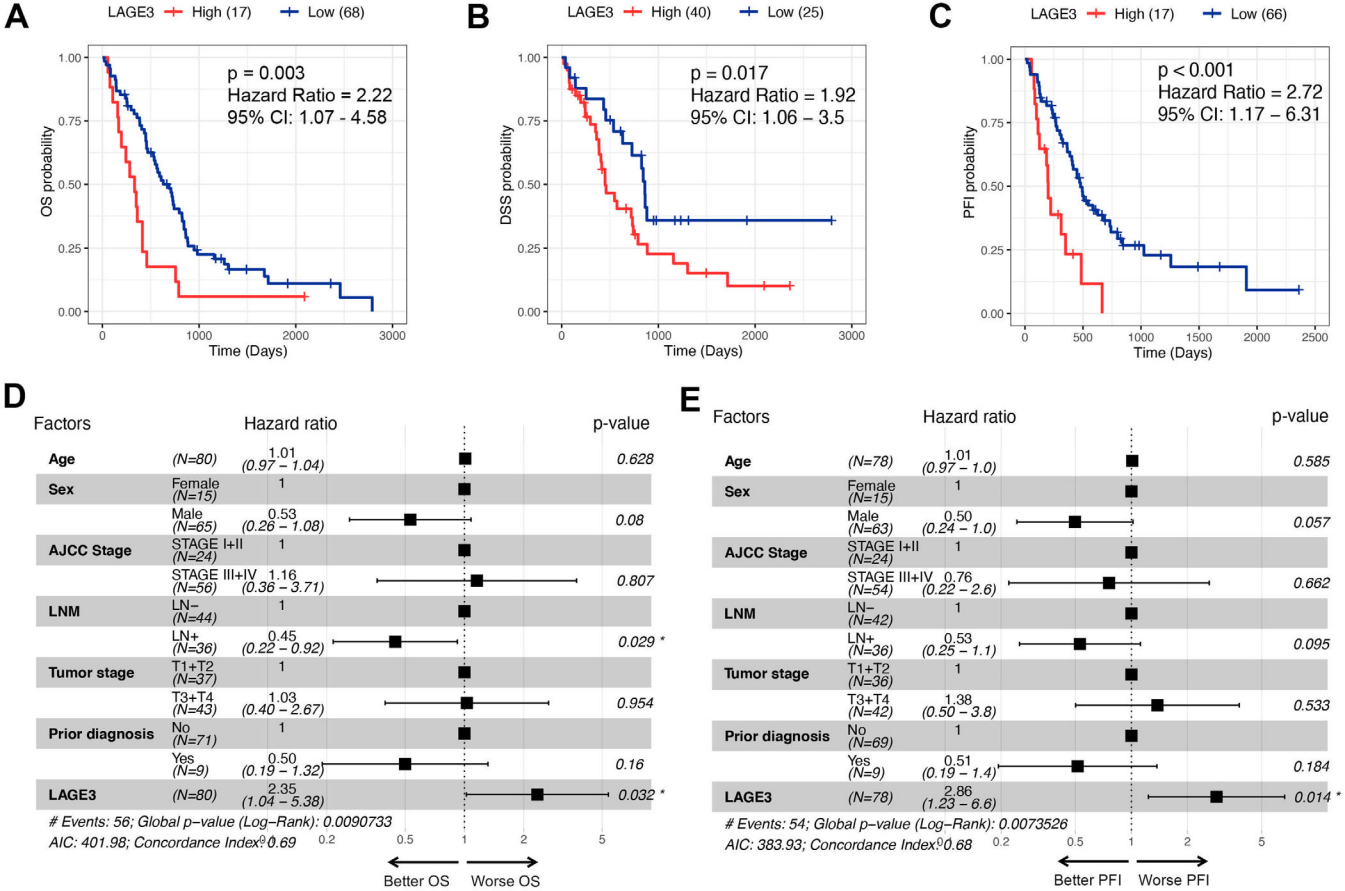
Survival analyses of LAGE3 and multivariate Cox regression of clinicopathological factors. **A‐C,** Survival analysis between the high‐LAGE3 group and low‐LAGE3 group of OS, DSS, and PFI. **D and F,** Multiple regression analysis of clinical parameters of OS and PFI. DSS, disease‐specific survival; OS, overall survival; PFI, progression‐free interval

We adopted ESTIMATE and xCell algorithms to estimate abundances of immune and stromal cells in the tumor microenvironment using RNA‐seq matrix data.^9^ The high‐LAGE3 group had a significantly higher immune score than the low‐LAGE3 group using ESTIMATE and xCell algorithms, while the stromal scores showed no significant difference between these two groups. Moreover, there is no significant difference between the high‐ and low‐LAGE3 groups of ESTIMATE score, tumor purity, and tumor microenvironment score (Figure S1). ImmuCellAI algorism was used to precisely explore the abundance of 24 immune cells using sequencing data.^10^ High expression of LAGE3 was correlated with a high proportion of B cells, CD8 T cells, macrophage, natural killer cells, Tex, Tgd, and Th2 cells. Conversely, naive CD4 cells were correlated with lower expression of LAGE3 (Figure 2A). In general, LAGE3 expression was positively correlated with a high proportion of immune cells. These results revealed that LAGE3 was significantly correlated with the immune scores and could regulate plenty of tumor‐related cell types in the MPM immune microenvironment. These results indicated that LAGE3 is significantly positively correlated with immune scores and may regulate different tumor‐related cell types in the immune microenvironment of MPM.

**FIGURE 2:**
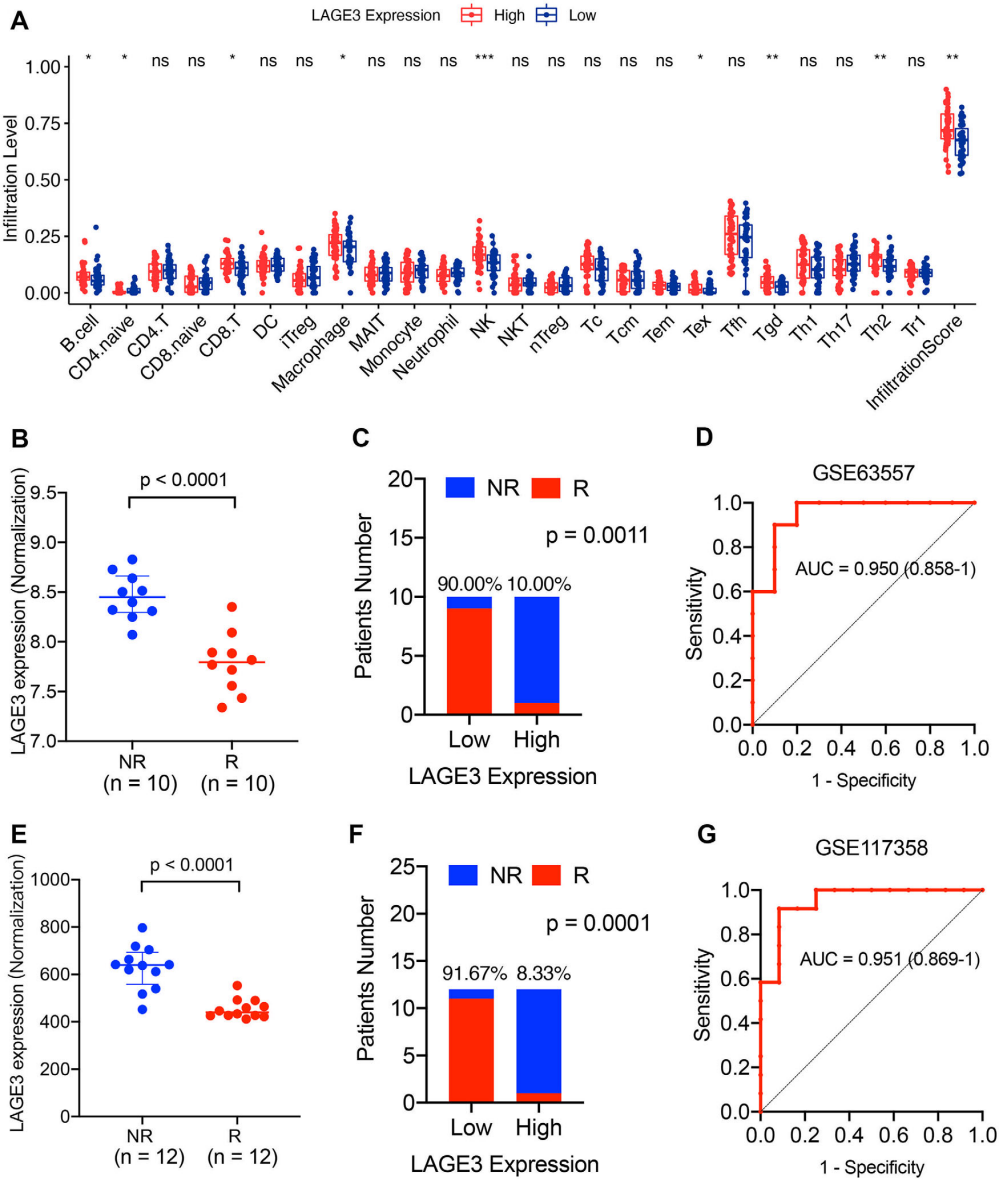
LAGE3 is predictive in immunotherapy response of MPM. **A,** The relative proportion of tumor‐infiltrating immune cells between the high‐LAGE3 group and the low‐LAGE3 group in MPM. **B,** The comparison of the LAGE3 expression between anti‐CTLA‐4 immune treatment response groups. **C,** The anti‐PD‐1 immunotherapy response in the high‐LAGE3 group and low‐LAGE3 group. **D,** Assessment of the predictive capacity of the LAGE3 and PD‐L1 measured by ROC curves in the GSE63557 cohort. **E,** The comparison of the LAGE3 expression between anti‐CTLA‐4 combined anti‐PD‐L1 therapies response groups. **F,** The anti‐CTLA‐4 combined anti‐PD‐L1 therapies response in the high‐LAGE3 group and low‐LAGE3 group. **G,** The predictive value of the LAGE3 measured by ROC curves in the GSE117358 cohort. MAIT, mucosal‐associated invariant T cells; NR/R, nonresponse or response; ROC, receiver operating characteristic; Tc, cytotoxic T cells; Tem, central memory T cells; Tex, exhausted T cells; Tfh, Follicular helper T cells

We subsequently investigated the predictive role of LAGE3 in the immunotherapeutic effect using the data from GSE63557 and GSE117358. Mesotheliomas with lower LAGE3 expression were more likely to respond to anti‐CTLA‐4 (Figure 2B, *P* < .0001) and antibodies against CTLA4 and PD‐L1 (Figure 2E, *P* < .0001). Compared with and the high‐LAGE3 group, the low‐LAGE3 group showed ninefold anti‐CTLA‐4 response rate (Figure 2C, *P* = .0011) and 11‐fold anti‐CTLA‐4 combined anti‐PD‐L1 response rate (Figure 2F, *P* = .0001). Surprisingly, LAGE3 alone enabled the classification of anti‐CTLA‐4 responders and nonresponders with a considerably predictive power (Figure 2D, area under the curve = 0.950). LAGE3 also achieved a predictive performance for those treated with CTLA‐4 combined PD‐L1 inhibitor (Figure 2G, area under the curve = 0.951). These results indicated that LAGE3 might have a strong predictive role in the immune microenvironment of MPM.

In conclusion, LAGE3 can potentially be used as an independent risk predictor for MPM patients. LAGE3 expression is related to the immune cells and can robustly predict the effect of immune checkpoint blockade therapy. However, the underlying mechanisms of immune microenvironment signaling pathways in MPM remain unclear, requiring further mechanistic experiments to verify. Knockout LAGE3 in the patient‐derived xenografts animal models may be a direction in future studies.

## CONFLICT OF INTEREST

The authors declare no conflict of interest.

## ETHICS APPROVAL AND CONSENT TO PARTICIPATE

The consent of ethics is waived because this study is based on public datasets.

## AUTHOR CONTRIBUTIONS

Xubin Dong and Jingjing Song designed the research. Xubin Dong, Jingjing Song, Minzhi He, Jiawen Sun, and Endong Tao performed the research and analyzed the results. Xubin Dong and Jiawen Sun wrote the paper. Xiaohua Zhang and Quan Li edited the manuscript and provided critical comments. All authors read and approved the final manuscript.

## FUNDING INFORMATION

Major Science and Technology Projects of Zhejiang Province; Grant Number: 2015C03052.

## Supporting information

Supplementary Figure 1 Comparison of tumor microenvironment scores with LAGE3 expression profiles in MPM. **(A)** Compare the immune score, stromal score, ESTIMATE score, and tumor purity calculated based on the ESTIMATE algorithm in the high‐LAGE3 and low‐LAGE3 groups. **(B)** Compare the immune score, stromal scores, and microenvironment scores calculated based on the xCell algorithm in the high‐LAGE3 and low‐LAGE3. **p < 0.01.

Supplementary Table 1 Univariate Cox regression of clinial pathological factors for overall survival in TCGA‐MESO patients.
Supplementary Table 2 Univariate Cox regression of clinial pathological factors for progression‐free intervel in TCGA‐MESO patients.

## Data Availability

Data and materials are all available upon reasonable request from the corresponding author.
